# The Impact of Institutional Formation on Firms’ Strategic Choices in Knowledge Development, Absorptive Capacity and Vertical Integration

**DOI:** 10.1007/s12116-022-09378-5

**Published:** 2022-11-18

**Authors:** Pradeep Kanta Ray, Anton Klarin, Sangeeta Ray

**Affiliations:** 1 University of New South Wales, Sydney, Australia; 2 Curtin Business School, Curtin University, WA 6102 Bentley, Australia; 3 University of Sydney, Sydney, Australia

**Keywords:** Institutional change, Strategic choices, Vertical integration, Human capital, Intangible assets, Institutional political economy, Russia

## Abstract

This study examines the impact of institutional shifts on the strategic choices of Russian firms. It proposes and tests hypotheses of how a shift from a weak to a strong institutional context is likely to affect firms’ knowledge accumulation, absorptive capacities and internalisation of operations. Using discriminant analysis, the econometric investigation demonstrates that firms tend to allocate greater resources towards improving their knowledge and absorptive capacity and make more efforts to vertically integrate—in line with improvements in the institutional environment. These investments ensure the survivability and competitiveness of firms in the long term. Furthermore, the study demonstrates that the long-term strategic orientation of firms goes hand in hand with rising resource allocations by the nation-state towards economic development. The findings align with the institutionalist political economy views that institutions are the ultimate overseers that allow the market to operate efficiently, especially in emerging market environments. The paper is also instructive to other developing economies about the need to strengthen their institutional environments, which supports the long-term orientation of firms and has a positive impact on economic development. The analysis does not take into account the impact of sanctions on Russian business and economy, post the annexation of Crimea and the armed conflict with Ukraine. Nor does it consider the impact of COVID-19 on the economy. As such, the study attempts to constitute an untainted comparison of two paths of transition on Russian firms—shock therapy, vis-à-vis, an institutional political economy approach.

## Introduction

Emerging economies often experience rapid and discontinuous shifts in their institutional environments. Among the major emerging economies, Russia underwent unprecedented and radical institutional changes in its quest to transform a weakening economy (Rutland 2013). Massive ‘institutional upheavals’ (Newman 2000) resulted from a sudden shift from a centrally planned to a liberalised market economy, then to a state-guided market economy. Russia’s pervasive institutional changes provide an ideal context for analysing strategic choices and business performance in a highly dynamic and turbulent environment. Although economic concepts such as the ‘valley of tears’ (Sachs 1991) or the ‘oligarchic property rights satisficing model’ (Braguinsky and Myerson 2007) may explain some elements of contrast in economic policy and influence on business performance, as of now, empirical research is somewhat sparse on how institutional changes influenced the workings of the Russian enterprises and how the relationship between institutions and product markets evolved through significant shifts in Russia’s economic strategy. Indeed, whatever limited research on Russian firms’ strategic choices exists (Peng 2001; Klarin and Ray 2021; 2019), they tend to either measure one short period of the transition or are qualitative, conceptual, or based on specific industry contexts. This is mainly due to the lack of reliable and consistent data and the difficulties in obtaining records (Hoskisson et al. 2000).

We adopt an institutional political economy perspective (IPE), analysing the effects of institutional formation on Russian firms’ strategic choices. A central thesis of the IPE literature is the importance of bringing institutions into the analytical core of our understanding of how markets function—that no market is free from other environmental influences that underpin its dynamics (Andreoni and Chang 2019; Lall 2013). Strategic choices—the process whereby firms decide on courses of action, using the agency of individuals and groups within firms to make choices—are made in response to environmental dynamism (Peng 2003; Montanari 1979; Klarin 2018). We posit a few plausible hypotheses that predict how Russian firms strategised in two dichotomous periods—in an institutional void versus an institutional formation phase. The first period of early institutional context marks Russia’s economic ‘shock therapy’ transition in the 1990s in the aftermath of the dissolution of the Soviet Union, from 1991 to 2006 (Rutland 2013). The second period began in 2007 (Baccini et al. 2018; Aslund 2004) and heralded the formalisation of the institutional context towards a guided market economy (Aslund 2009; Klarin and Ray 2021) (see Table 1 for a list of select reforms). The approach in the second phase is characterised by public–private partnerships (PPPs) in clusters, such as Skolkovo, which serves as the most prominent Russian innovation incubator (Medvedev 2017); various programmes that support high-tech industries, including aviation, electronics, medical and pharmaceutical, defence and shipbuilding; and the increasing emphasis of policymakers to transition to a digital economy (Ministry of Industry and Trade of the Russian Federation 2017). This stance contrasts with the earlier neoliberal approach of minimalist involvement.

**Table 1:** Russian institutional voids (1990s) and institutional formation (2000s)

Institutional voids	Institutional formation
1991–2000	Formal institutional measures, 2001 onwards
Voids related to lack of accounting standards (credibility enhancers)	• IFRS and International Accounting Standards reporting requirements and procedures (2002); • Federal Law On Credit Histories (State Duma) and introduction of credit bureaus (2005)
Voids related to lack of regulatory oversight—tax evasion	• Tax regulation and more effective enforcement (from 2001), including small and medium-sized enterprise tax simplification—either 6% on revenue or 15% on profit, a flat rate of 13% personal income tax and reduction of corporate tax from 35 to 24%
Voids created by policy dysfunction—decentralisation of power	• Laws passed to consolidate power centrally (Bahry 2005): On Modifications and Additions to the Federal Law On General Principles of the Organisation of the Legislative (Representative) and Executive Bodies of State Power of the Subjects of the Russian Federation (State Duma) and On the Formation of the Federation Council of the Federal Assembly of the Russian Federation (State Duma) • Local government checks and balances system introduced—On General Principles of Organisation of the Local Self-Government in the Russian Federation 1999 (State Duma) • Alignment of laws and regulations federally—Civic Chamber (established in 2005)
1991–2000	Normative measures to fill institutional voids, 2007 onwards
Financial system voids	• Various laws, including On Consolidated Financial Statements Law 2010 (State Duma), stipulate that public companies must adhere to IFRS
Adjudicators voids related to judicial and enforcement systems failures	• Government support programme—‘Development of the Court System for 2007–2011’, to improve the efficiency of the judicial system • Better control and incentive mechanisms for judiciary and enforcement (Solomon 2008)
Lack of non-government organisations	• Non-government organisations related to key strategic industries are encouraged, while those attributed to foreign investors face various pressures (Govorun, Marques, and Pyle 2016)
Voids related to lack of innovation ecosystem—neglect of science and technology	• As an example, the Skolkovo Innovation Center initiative is designed to create a sustainable innovation ecosystem and foster a culture of entrepreneurship to support the development and commercialisation of advanced technologies in the Russian Federation and beyond
1991–2000	Cultural-cognitive measures to fill institutional voids, 2010 onwards
Trading is the prevalent business model	• Increased sentiments for economic diversification away from the natural resource sector and sovereignty (early 2010s), including propagation of science and technology

It is well to state upfront that the analysis presented does not purport to be a definitive word on the effects of institutional change on Russian firms’ strategic choices, nor is it intended to be a statement on the current institutional context or geopolitical situation facing Russia. Our paper goes only so far as comparing two dichotomous periods pre- and post-reforms, up to 2014. Admittedly, this restricted window of investigation, 2007–2014, has limitations. Nonetheless, our study is the first to present an empirical model of analysis of the changes in Russia’s institutional landscape and the strategic choices of Russian firms between 2007 and 2014. Adopting discriminant analysis (DA) and regression analysis, we explore the impact of institutional formation on firms’ strategic choices (2007–2014).

Our study is novel in its contribution to understanding the co-evolution of institutions and the workings of markets in transition economies. We adopt an institutional political economy perspective, analysing the effects of institutional strengthening on Russian organisations’ strategic choices. Understanding the influence of Russian institutional change contributes to the literature by providing valuable insights into the varying transitions of the other fourteen ex-Soviet transition republics that were part of the largest union of nation-states in the twentieth century. Although these countries are distinct in their development and institutional environments, the Soviet-era ethos that emphasises equality, socialisation of property and collectivism remains strong in these ex-Soviet nation-states (Gevorkyan 2013; 2018; Puffer and McCarthy 2011; Yegorov 2009). The enduring institutional similarities between Russia and other ex-Soviet republics allow comparisons across these countries.

Essentially, this study suggests that institutional strengthening positively impacts the strategic choices of emerging market firms. With newly introduced regulations and normative institutions, firms begin to invest heavily into long-term asset creation, including knowledge development and codification, measured through intangible asset accumulation; absorptive capacity as reflected in human capital investments; and internalisation of activities or the vertical integration of operational capacities. This is in sharp contrast to firms engaging in short-term trading and abstaining from knowledge accumulation activities due to voids and uncertainties in the institutional environment during the shock therapy transition of the 1990s. A novel methodology is used in this study to derive these findings. Adopting quantitative discriminant and panel analyses, we understand the real effect of reforms on Russian organisations’ strategic choices.

This paper is structured as follows. We first introduce the neoliberalism approach to transition as a shock therapy prescription, as per the Washington Consensus recommendation adopted in the 1990s in the majority of ex-Soviet countries, followed by the institutional political economy alternative that Russia adopted in the second phase of transition. Section 2 further provides the research background of the two stages of the Russian shift and outlines the hypotheses to be tested in this study. Section 3 is the methodology section and outlines data sources, variables and the analysis undertaken in this study. The findings and discussions based on testing the proposed hypotheses are followed by the conclusion and implications of the study.

## Literature Review and Research Background

### Theoretical Underpinnings: Neoliberalism and the Institutional Political Economy Alternative

In the 1990s, following the collapse of the centrally planned command and control system, the erstwhile post-Soviet socialist states liberalised their economies to usher in the prospect of liberal market capitalism. Neoliberalism, the dominant paradigm in the Washington Consensus, advocates unconditional support for unrestricted market freedoms (Williamson 2009). The neoliberal school embodies the ideological premises, value judgements and assumptions of many modern development theories today (Lall 1994; Evensky 2005). Neoliberal libertarians believe in the efficiency of free markets and advocate complete deregulation of the economy. Accordingly, the ideal policy for any less developed country is to embrace wholesale economic liberalisation—leading to a laissez-faire market economy that opens up trade and encourages efficient industrial organisations from developed countries to improve competition and output (Stigler 1988). Such was the view before and during the liberalisation of the Russian and other ex-Soviet states in the 1990s.

However, sudden liberalisation can potentially unleash unpredictable changes in an institutional context. Unsteady reforms can potentially make it difficult for firms to operate coherently (Newman 2000). Under extreme changes, the operating conditions are too chaotic and uncertain, such that the institutional context no longer provides organising templates, models for action and known sources of legitimacy (Woldesenbet 2018). Indeed, large-scale changes are known to cause ‘institutional voids’ that are described as weaknesses in the transactional guidelines between buyers and sellers and also include voids in other formal institutions such as government and related agencies (McCarthy and Puffer 2016). Under rapid and extreme changes, it is difficult to learn from experience because past events have little value as a guide for future action, as cause-effect relationships become ambiguous and cannot be readily discerned (Mahoney and Thelen 2010). Under this large-scale and discontinuous change, the pace of dismantling older institutions typically does not coincide with the construction of new institutions, resulting in a deep chasm between old and new institutions and profound uncertainty (Newman 2000; McCarthy and Puffer 2016). Due to the messiness and chaos of such economic transition, economic actors cannot make sufficient sense of their situation or act coherently (Thagard 2000).

Institutional political economists argue it is somewhat dubious to assume any country—especially a developing one—can fully benefit from sudden liberalisation without the supporting role of policy and non-market institutions, both formal and informal (Chang 2002b; Lall 2006). IPE scholars hold that markets originate in institutions and that institutions are deliberately engineered (Fioretos et al. 2016). Furthermore, the IPE perspective advocates that a guided market economy is necessary for economic development, especially in developing countries (Andreoni et al. 2019).Proponents abide by the core principles of institutional economics (North 1990) and hold that economic and market domains are not isolated but essentially interwoven with political and social environments. Thus, according to IPE scholars, markets are essentially a political construct and are themselves considered institutions. The catch-up of East Asian countries that adopted a guided market economy approach reveals a trend of robust policy framework, driving investments in favour of strategic industries and the grafting of capabilities in innovation through learning from an export-oriented industrialisation strategy (Chang 2002a; Andreoni and Chang 2019). Critics hold that the Russian shock therapy approach had unintended economic effects (Sachs et al. 1994; Marangos 2003) compared to China and India, which followed a gradualist, step-by-step process.

### Research Background: Business Environment During the Russian Transition

Russia underwent a cataclysmic transition overnight from a command and control system into a liberal free market economy with the end of the Gorbachev era. Russia’s sudden shift into a neoliberal style of governance in 1991 was envisioned on the premise that wholesale liberalisation would help it to develop faster by welcoming foreign investment and technology under open trade and investment policies (Balassa 1988; Krueger 1990; Ulusoy and Taş 2017; Ettlinger and Hartmann 2015). Russian radical reformers inspired by the International Monetary Fund believed that markets are ‘natural’ and ‘spontaneous’ social orders that can supposedly flourish and develop best in the absence of any intervention. The assumption that markets would somehow materialise and institutions would present in the natural course of things was not only the premise of the new liberalist thinking but also formed much of the basis of economic policies (Yeltsin 2000; Lall 1996). This view has been widely criticised in literature (see, for example, Stiglitz 1999).

After the sudden liberalisation, state monopolies and industrial output collapsed due to the rapid breakdown of the Soviet-era value chains and networks. Most organisations were unable to sustain operations, resulting in mass closures and industry disruptions (Murphy et al. 1992; McFaul 1995). The distinctive features of the newly fashioned neoliberal Russian business systems included start-up organisations (Peng 2001), networking (Michailova and Worm 2003; Puffer et al. 2010) and opportunistic practices (Volkov 1999), taking the comparatively easy route of importing and arbitrage-based trading over true reform of domestic markets and industry (Rutland 2013). The unpredictable new structure did little to regulate corporate governance, accounting procedures, laws on disclosure and payment of dividends. This resulted in a lack of information and rights for investors (Black and Tarassova 2003). The fledgling stock markets had little regulatory oversight, resulting in the dominance of directors and bankers, not entrepreneurs, and insider trading was the norm rather than the exception (McFaul 1995; Guriev and Rachinsky 2005).

The ownership of large state-owned firms in the communist era was transferred to the oligarchs (Guriev and Rachinsky 2005; Rutland 2013; Goldman 2004; Annushkina 2013; Fidrmuc and Gundacker 2017). Some oligarchs successfully exerted their monopolistic power in a distorted market, and there is evidence to suggest that there was little innovation or long-term investments. Moreover, wholesale liberalisation resulted in the growing inequality and the related problems of the early Russian transition, ultimately leading to the failed neoliberal experiment (for a detailed account of the economic ills associated with oligarchic rule in 1990s Russia, see Fortesque (2006)). In other words, barring a few oligarchies, the vast majority of Russian firms suffered the blight of a dysfunctional institutional context that prevented them from making long-term investments (Goldman 2004; Aslund 2004; Guriev and Rachinsky 2005).

Considering the gaps within formal institutions due to the large-scale transition, Russian organisations relied on informal institutions to the detriment of long-term investment practices (Granville and Leonard 2010). In these circumstances, organisations in emerging markets often rely on political networks for strategic sensemaking of unfolding environments (Klarin and Sharmelly 2021), which was also the case in Russia (Klarin and Ray 2019). Deep uncertainties and lack of confidence led most Russian business leaders to favour short-term profits at the expense of long-term investment in their production facilities and innovation (Filippov 2011). Additionally, structural transformation problems at the beginning of the 1990s and severe financial difficulties prevented the Russian Government and the newly liberalised organisations from supporting scientific and innovation-related investments and strategic policies (Dyker 2001). Instead, the opening of international borders facilitated trade and intermediary relations. Organisations focused on commercial activity with instantaneous profits from ever-increasing demand (Kvintradze 2010). A large section of the business elite (led by oligarchs) engaged in various quasi-legal practices to circumvent uncertainties and transaction costs—and successfully amassed fortunes almost instantly (Guriev and Rachinsky 2005; Cheloukhine and King 2007).

As observed previously, the literature suggests that the shock therapy ‘big bang’ approach based on liberalisation, privatisation and deregulation as pillars of radical reform strategy lacks an appreciation of the ‘conditions required for it to work effectively’ (Stiglitz 2000, 201). The chaos of an economic transition does not allow economic actors to make sufficient sense of their situation such that they can act coherently (Puffer et al. 2010; Thagard 2000; Weick and Whitener 2005). The unpredictable new institutional structure cannot regulate corporate governance, accounting procedures, laws on disclosure and other essential functions—leading to the mounting industrial chaos that occurred in the period of early institutional formation in the nascent Russian Federation.

The second period in the early 2000s, which we term ‘guided market capitalism’, saw a progress towards establishing regulatory institutions to guide businesses. After a decade of chaos, it was abundantly clear that markets were not ‘natural’ and ‘spontaneous’ social orders and that there needed to be an institutional system to regulate and guide business (Rutland 2013; Aslund 2004; Lall 1996). Gerasimenko (2012) notes that institutional reforms in the regulatory domain included court system reforms, effective enforcement of new tax regulations, signing of the UN Convention Against Transnational Crime and Corruption, the passing of laws on money laundering, guidance provided on acceptable corporate conduct and institutionalisation of civil service practices. Additionally, the adjustment of capital markets and adherence to International Financial Reporting Standards, among other changes, helped develop a consolidated formal institutional environment (see Table 1 for a list of select reforms).

The Russian Government also implemented new policies to promote innovation, even as old institutions were reformed (Filippov 2011). Concurrently, Russia demonstrated commercial policy recalibration by changing its priorities towards import-substituting industrialisation. ‘The Concept of Long-term Socio-Economic Development of the Russian Federation up to the year 2020’ included investments in human and physical capital and infrastructure between 2009 and 2020 (Gerasimenko 2012; Government of the Russian Federation 2011; Gokhberg and Kuznetsova 2011). This was followed by successive government programmes, including the facilitation of ‘high-tech’ sectors, such as the aircraft industry and propulsion engineering, spacecraft and rocket industry, radio electronics industry, nuclear energy and information and communication technologies (Skhvediani and Sosnovskikh 2020). With institutions strengthening, Russian organisations shifted their strategy towards manufacturing investments in plants and machinery and, later, investments in innovation (Gokhberg and Kuznetsova 2011; Sosnovskikh 2017).

However, even at present, many idiosyncratic pressures persist, and new ones have arisen since the 2000s, including corporate raiding, power networks and many informal institutions related to the current power structure (Gans-Morse 2013; Osipian 2018; Ledeneva 2013; Viktorov and Abramov 2022). Furthermore, an in-depth study of the Russian industrial clusters demonstrates that the idiosyncratic cultural, governmental and attitudinal aspects prevent Russian industries and firms from fully developing and integrating into the global market (Sosnovskikh and Cronin 2021).

### Hypotheses Development: Changes in Strategic Choices in Response to the Institutional Formation

Higher levels of institutionalisation can usher in increased foreign competition motivated to invest directly in the domestic environment because of improved intellectual property rights protection (Dyker 2001; Gerasimenko 2012). Consequently, competition increases for domestic firms exposed to new foreign competition and imports (Sigurdson 2000; Wang et al. 2018). Firms must, therefore, improve efficiency, productivity, product quality, customer service and product development. Hence, there are incentives for improved technology and innovation capabilities (Geske Dijkstra 2000; Feinson 2003; Tomizawa et al. 2019).

Drawing from the literature, this study posits a few fundamental hypotheses as to how a shift from an institutional void to an institutional formation era may be accompanied by firms’ deliberate change towards long-term orientation, marked by increased resource commitments towards the creation of intellectual property in the form of codified knowledge (Mahoney et al., 1992; Pisano 1991), the development of a specialised human capital base (Dakhli and De Clercq 2004; Nyberg et al. 2014) and internalisation of value chain activities (Brocas 2003).

### Strategic Choices: Investment in Knowledge Development and Codification

There is growing support in the literature to extend the resource-based approach into a knowledge-based view that proposes that knowledge is an essential part of the strategy toolbox of firms trying to catch up in the global economy (Eisenhardt and Martin 2000; Teece 2014). Organisations’ knowledge bases put intellectual capital at the front and centre of advantages that secure the long-term competitiveness of firms and countries (Bharadwaj 2000; Inkinen 2015). Knowledge, defined elsewhere as ‘intellectual capital’, is the only appreciable asset of the firm, while most other assets depreciate over time, including machinery, buildings, industrial plants and equipment (Ulrich 1998; Andreeva and Garanina 2016). An essential indicator of intellectual capital is a firm’s codified knowledge base, which includes its current state of publications, training, techniques, devices and specialised libraries can be taken (Cohen and Levinthal 1990; Matthyssens et al. 2006). Other literature argues that a firm’s codified knowledge base is represented by reputation, relational assets, patents, copyrights, trademarks, rights to access technology resources, goodwill and distributional infrastructure (Bharadwaj 2000; Dunning and Lundan 2008; Delios and Beamish 2001).

**Fig. 1:**
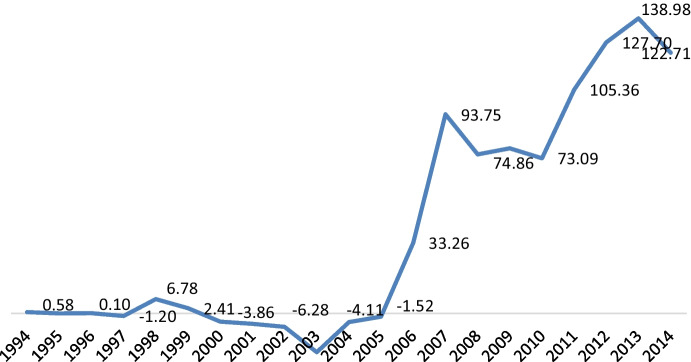
**Investments in knowledge codification by Russian firms.** *The data points were calculated based on cumulative logarithmic firm data from the Compustat database

During the shock therapy years, Russian firms neglected long-term investment practices in knowledge accumulation, preferring to look for short-term profits through imports and trading activities. Due to the voids in formal institutions, this period was marked by a near-total absence of investments in knowledge accumulation. With institutions forming (see Table 1), signs of long-term investment behaviour are clear (Chadee and Roxas 2013; McCarthy et al. 2014). Seeing the need for strategic diversification of the economy, Russia took the lead in developing key strategic sectors. In the institutional formation period of the 2000s, Russian firms invested more in the codification of knowledge to provide long-term competitiveness (see Fig. 1). This is because institutional formation that protects intellectual property presents greater incentives for firms to codify what they ‘know’ due to lessened fears of patent breaches and allows them to leverage that explicit knowledge for long-term strategic advantage (S. Ray 2013; S. Ray and Ray 2021). Once formal institutions began forming, firms shifted their strategy from gaining quick returns from trading and arms-length transactions to devoting greater resources to knowledge development and exploitation (Knack and Keefer 1995; Nelson 2008; Lall 2013).

Hence, a plausible hypothesis would be that institutional formation will positively influence investments by codifying a firm’s knowledge base:

- **Hypothesis 1:** The propensity of firms in transition economies to invest in knowledge codification will be higher in the institutional formation phase.

### Strategic Choices: Investment in Absorptive Capacity

The formation of institutions provides stimuli to technical change and investment in the workforce (Nelson and Nelson 2002; S. Ray 2013; Lundvall 2016). Stable and effective market institutions encourage sustained investment in human capital and technologies (Granville and Leonard 2010; Acemoglu and Robinson 2002) and recruitment of high-quality human capital. Evidently, a firm’s ability to identify, assimilate and exploit extramural knowledge from the environment is primarily a function of the level of prior related knowledge or absorptive capacity. A firm’s absorptive capacity will depend on the absorptive capacities of its individual members (Cohen and Levinthal 1990; S. Ray 2013) or human capital. Firms attract high-quality human capital, often from competitors for exploratory innovations, by offering high salaries (S. Ray 2013; Chen and Lin 2004; Cirillo et al. 2017; Krugman 1994; S. Lee and Meyer-Doyle 2018).

The resource-based perspective dictates that absorptive capacity may constitute the core of competitiveness due to its features, including inimitability, rarity, value and non-substitutability (Nyberg et al. 2014). Investments in absorptive capacity, including salaries paid, training and various job enhancements, create a more robust knowledge base and, consequently, improve a firm’s competitiveness (Dakhli and De Clercq 2004; S. Ray 2013; McGuirk et al. 2015). Nahapiet and Ghoshal (1998) refer to intellectual capital as a social collective’s knowledge and knowing capability.

- **Hypothesis 2: ** The propensity of firms in transition economies to invest in absorptive capacity will be higher in the institutional formation phase.

### Strategic Choices: Investment in Vertical Integration

In a weak institutional context, businesses often focus on arbitrage opportunities such as trading, where profits are instantaneous (Kvintradze 2010). For example, sudden liberalisation led companies in Russia to become short-term oriented, engaging in trading and asset stripping companies while investors stayed away (Hitt et al. 2004; King 2002). Institutional formation motivates firms to invest in quality and optimise supply chain management (Silvestre 2015). This often entails investments in internalising operations within firm boundaries by integrating most upstream and downstream value-adding activities in-house (Turkulainen et al. 2017; Macher and Richman 2008). Supply chain volatilities and quality uncertainty also lead firms to vertically integrate their operations. Vertical integration enables industries to understand the process technologies involved and grasp the underlying technological principles behind those processes (Ettlie and Reza 1992; Helfat and Campo-Rembado 2016). Generally speaking, learning tacit elements of new technology requires greater internalisation due to asset specificities (P. K. Ray et al. 2004). A vertical integration strategy helped many world-class producers such as Ford and Daimler-Benz learn complex technologies and lead exports (Kumpe and Bolwijn 1988). Business executives often defend vertical integration as there may be economies of internal production that prevent market failures caused by externalities and imperfect information (Lundvall 2016; Teece 2014). Vertical integration also provides a cushion against volatility in exchange rate fluctuations and supply bottlenecks due to infrastructural problems (Bandarenka 2016). The benefits of vertical integration come from the greater control afforded to firms over access to inputs and the cost, quality and delivery times of those inputs (Harrigan 1984; Dorobantu et al. 2017).

Given what is known from theory and empirical data regarding investments made by Russian firms, the following hypothesis is proposed:

- **Hypothesis 3:** The propensity of firms in transition economies to invest in vertical integration will be higher in the institutional formation phase.

The empirical literature is limited in rigorously assessing the strategic choices of Russian firms during the two different transition paths undertaken in the country since the 1990s. This study draws from the most extensive dataset available of Russian firms and their investment behaviour in the two periods. We hypothesise that more robust government intervention in creating institutions results in firm investment into knowledge development and codification, absorptive capacity and vertical integration through the internalisation of activities.

## Model, Data and Variables

The preliminary empirical examination of the evolution of Russian firms indicates that a post-reform institutional context (2007–2014 period) positively impacts Russian firms’ strategic choices insofar as how they allocate resources towards knowledge codification, vertical integration and the development of absorptive capacity. Accordingly, we predict that firms’ strategic decisions would undergo a positive shift in the institutional formation phase from 2007 to 2014. To test these hypotheses, we compare strategic choices in two specific periods—the institutional voids phase (1999–2006) and the institutional formation phase (2007–2014)—using a multivariate framework via the application of Fisher’s DA using SPSS (version 26) and STATA (version 16). Fisher’s discriminant function analysis has been extensively used in the economics and management literature (Kumar 1991; Arya et al. 2000; Lim et al. 2006; Subramaniam and Hewett 2004; Tsang 2002) where the research problem essentially entails discriminating between two different entities. This technique is robust, handles categorical and continuous variables and has coefficients many find easier to interpret (Hair et al. 1998).

Following the DA technique, the discriminating variables, namely knowledge codification, absorptive capacity and vertical integration, can be compared and contrasted by the following equations corresponding to two dichotomous phases:

Institutional Voids Phase_0_ 1999–2006:

$${Y}_{1}={c}_{1}+{a}_{1}{x}_{1}+{a}_{2}{x}_{2}+..+{a}_{n}{x}_{n}$$

Institutional Formation Phase_1_ 2007–2014:

$${Y}_{2}={c}_{2}+{b}_{1}{x}_{1}+{b}_{2}{x}_{2}+..+{b}_{n}{x}_{n}$$

where *a* and *b* are discriminant coefficients for the two respective phases, *x* is discriminating variables (knowledge codification, absorptive capacity and vertical integration) and *c* is a constant.

Discriminant functions involve fitting linear discriminant score functions based on observed data on several discriminating variables for individual cases whose group membership is known. Thus, if the available variables are *x*_1_, *x*_2_, *x*_3_, … *x*_*n*_, the best set of variables is the one that will produce maximum distance in space among the group centroids ¯*X*_1_ ¯*X*_2_, ¯*X*_3_, …. ¯*X*_*n*_. If a significant difference among the groups is found, DA then finds which linear combinations of the explanatory variables (independent predictor variables) give the most considerable means differences between the existing groups to best discriminate between the groups defined. The classification functions can then estimate the probability of a particular firm’s membership in each group. As mentioned above, in the equations, the panel of Russian firms is stratified into specific groups (phases), the first group coinciding with the institutional voids phase (1999–2006) and the second group (2007–2014) coinciding with the institutional formation phase. The test of the equality of group means is conducted via Wilks’ Lamda, which indicates how well each function separates cases into groups, in this case, phases (see Table 6). Finally, DA calculates what percentage of cases is correctly classified—through a classification matrix that confirms or refutes whether the model based on a priori groupings is accurate (see Table 5).

To further test the robustness of the results, we ran a multi-level analysis using pooled models using the fixed effects model (see Table 7), incorporating panel data in testing the effect of institutions on the level of codified knowledge, absorptive capacity and vertical integration of firms (Maddigan 1981; Robertson and Langlois 1995). Appendix provides more information on the methodology used.

We do note that true to the nature of developing countries, the institutional formation would probably continue beyond 2014. However, the Russian annexation of Crimea in 2014 resulted in a wide range of sanctions that consequently changed the Russian business environment. We thus abstained from analysing Russian firms post sanctions as it would inevitably affect comparing the weaker institutional environment of the first measured phase to the institutional formation phase prior to 2014.

### Data Sources

For this study, we utilised data from Compustat Global. Compustat Global contains financial statement information on all listed firms in Russia from 1994. This database is an important source that collects data from various countries and is widely recognised as a reliable source in the fields of accounting, finance, strategy and management (Bharadwaj 2000; Boubakri et al. 2013). Compustat data surveys the same publicly listed firms each year. Firm-level data in Compustat represents 98% of total market capitalisation. We utilised all listed Russian firms’ data in Compustat to constitute the population. A panel of 282 listed firms from 1999 to 2014 was generated, yielding 2917 total firm-year observations. The entire dataset of firms was then stratified into two periods: 1999–2006 (institutional voids period) and 2007–2014 post-Skolkovo (institutional formation period). Using these stratification criteria, the SPSS statistical package yielded an unbalanced sample of 102 firms in the first period and 282 firms in the second period, with the second period sample including 98 firms from the first period sample (four firms in the first period sample did not survive through to the second period).

### Variables in the Firm-Level Analysis

#### Knowledge Codification

Codified knowledge is accumulated and embedded in firms’ intangible assets, consisting of patents, blueprints, databases, manuals and scientific publications (Nelson and Winter 1982). Expenses incurred by firms in establishing, maintaining and updating these forms of intangible assets can, therefore, be used as a proxy for firms’ efforts in codifying knowledge. In this study, logarithmic expenditure in developing intangible assets was used to measure the codification of knowledge by a firm. We used the log transformation simply because intangible assets are a stock measure, not flow, and part of a firm’s balance sheet.

#### Absorptive Capacity

Absorptive capacity is signalled by the quality of human capital and is captured by employees’ individual and team-specific abilities (Bresnahan et al. 2002; De and Dutta 2007). Staff expenses express the measurement of a firm’s commitment to human capital in the accounting context (Chen and Lin 2004). More specifically, salaries and wages are used as an indicator to measure the intensity of investment into human capital (De and Dutta 2007). Firms with higher quality human capital are likely to incur high expenditures on salaries. In this study, salaries paid by firms as a proportion of sales were used to measure the firms’ efforts to develop a high-quality human capital base. Investments in staff expenditures are measured yearly and, therefore, constitute a firm’s inputs during the measured period.

#### Vertical Integration

The level of vertical integration is reflected by what extent firms perform value-adding activities in-house, as opposed to depending on suppliers outside the boundaries of firms. Previous studies have employed a ratio to measure vertical integration, such as value added over sales, that should move consistently with the number of processes performed by a firm or an industry. This strategy has been employed, with some variation, by Adelman (1955), Gort (1962), Laffer (1969) and Tucker and Wilder (1977). Rumelt (1986) suggested a ratio whereby the vertical integration of a firm can also be reflected by the percentage of the total product that is part of a firm’s vertical chain. The present study adopted value added as a percentage of sales for ease of calculation (Maddigan 1981). The PIMS database includes vertical integration measures as absolute and relative. The absolute measure is value added as a percentage of sales for each business unit.

## Variables in the Multi-Level Analysis

We conducted a multi-level analysis to test the robustness of the firm-level analysis. In addition to temporal bracketing (institutional voids versus formation phase) as an independent (dummy) variable, we introduced three additional nominal variables representing the quality of institutions in firms’ strategic choices. The additional institutional variables include scientific publications, inbound foreign direct investment (FDI) and gross domestic research and development (R&D) expenditure (Teece 1985; Zhang and Song 2000).

The output of scientific publications, patents, copyrights and other intellectual property—the scientific output—signifies the innovation output of the economy and, hence, its institutional quality (Pratikto 2013). Increasing the innovation output of the economy strongly impacts a firm’s choice to invest in innovation, vertical integration and absorptive capacity. The literature also suggests that increased national-level expenditure on R&D exerts institutional influences on the focal firm’s autonomous investments in innovation. FDI inflows measured as a percentage of gross domestic product (GDP) are used as a standard proxy of confidence for institutional quality in the country. We included accepted standard controls, including firms’ revenue and asset base. The dependent variables remain as before: knowledge codification, absorptive capacity and vertical integration (Table 2).

**Table 2:** Definitions of dependent, independent and control variables

Firm-level variable	Variable measure
Knowledge codification	Intangible assets—codified knowledge measured by the log of intangible asset expenditure in USD (Intangible_Assets)
Absorptive capacity	Human capital—absorptive capacity of human capital is measured by salaries, wages, training expenses and other emoluments (Human_Capital) as a percentage of sales for each business unit
Vertical integration	Value added—vertical integration (internalisation) is measured by the log of value added by the firm in USD (Value_Added). The PIMS database includes vertical integration measured as value added as a percentage of sales for each business unit
Institutional variable	Variable measure
Institutional voids: 0 Institutional formation: 1	Institutional voids period (Voids_0_) 1999–2006 (adjusted for the lag effect of the implementation of the reforms) and the institutional formation period (Formation_1_)_2007–2014
Scientific output	Number of scientific publications, patents, copyrights and other intellectual property (Log)
Foreign direct investment (FDI)	FDI inflows (cumulative) into Russia in the respective periods (% of GDP)
Research and development (R&D)	Gross domestic expenditure on R&D (% of GDP)
Firm-specific control variables	Firm-specific variable measure
Return on assets	Return on assets of the firm
Revenue	Total revenue of the firm

## Findings and Discussion

The results below reflect how accurately the difference in context—that is institutional voids (1999–2006) versus institutional formation phases (2007–2014)—is reflected in Russian firms’ strategic choices. In Table 3, the mean differences are in accordance with the predictions in respect of all three variables in question; knowledge codification, absorptive capacity and vertical integration are higher in the institutional formation phase than in the institutional voids phase (0.69 > 0.13, 12.13 > 3.82 and 2.46 > 1.75, respectively). The test of equality of group means (see Tables 4). Test of equality of group meansVariableWilks’ LambdaFSig. of F to removeKnowledge codification (intangible assets)**0.9896.570.10Absorptive capacity (human capital*)0.97415.760.00Vertical integration (value added)*0.97514.980.00^5^) indicates a clear dichotomy between the two phases insofar as how they affect the discriminating variables of knowledge codification, absorptive capacity and vertical integration. Therefore, the institutional formation phase appears to positively influence a firm’s human capital, intangible assets and vertical integration. Table 5 (classification matrix) confirms that 77.5% of the groups were correctly classified, increasing the plausibility of the predictive accuracy of our model. We now discuss the results of Table 6 in detail, in turn.

**Table 3:** Means and standard deviations of variables

Variables	Institutional voids context 0 (1999–2006)		Institutional formation context 1 (2007–2014)	
	Mean	*SD*	Mean	*SD*
Knowledge codification (intangible assets)**	0.13	1.27	0.69	1.00
Absorptive capacity (human capital*)	3.82	8.28	12.13	10.15
Vertical integration (value added)*	1.75	0.88	2.46	0.83

**Table 4:** Test of equality of group means

Variable	Wilks’ Lambda	*F*	Sig. of *F* to remove
Knowledge codification (intangible assets)**	0.989	6.57	0.10
Absorptive capacity (human capital*)	0.974	15.76	0.00
Vertical integration (value added)*	0.975	14.98	0.00

**Table 5:** Classification matrix

	Voids: 0 Formation: 1		Predicted group membership: Voids: 0 Formation: 1	
%	0	69.6		30.4
%	1	22.2		77.8

77.5% of originally grouped cases were correctly classified

**Table 6:** Fisher’s linear discriminant functions

	Institutional voids (1999–2006): 0	Institutional formation (2007–2014): 1
Knowledge codification (intangible assets)**	− 2.02	− 1.90
Absorptive capacity (human capital*)	4.11	5.06
Vertical integration (value added)*	0.02	0.11

Hypothesis 1 posited that institutional formation (2007–2014) would spur managerial confidence in the ability of institutions to protect property rights (OECD 2011; Gans-Morse 2013) and encourage the importance of forming a firm’s codified knowledge base (Tomizawa et al. 2019). However, the results in Table 6 suggest that knowledge codification is negatively affected in both the institutional voids and institutional formation phases and, thus, is not a good predictor of group membership either way. This result could also be an artefact of an enduring lack of managerial confidence in the ability of institutions to protect property rights or simply the fact that institutional changes take a long time to instil managerial confidence to make investments in knowledge codification. Hypothesis 1 will need to be tested further in the robustness checks (see Table 7) to come to any definitive conclusion regarding its plausibility.

**Table 7:** Results of pooled regressions

Variable type	Measures	Knowledge codification	Vertical integration	Absorptive capacity
Control variables	Revenue	− 0.01 (0.06)	0.80*** (0.02)	.029 (0.09)
	RoA	− 0.61* (0.32)	1.67*** (0.09)	− 0.75 (0.34)
Institutional variables	Scientific output	2.54* (1.43)	1.07*** (0.27)	2.49* (1.46)
	FDI	0.05 (0.05)	0.00 (0.01)	.05 (0.05)
	R&D expenditure	− 1.05 (1.36)	0.67*** (0.25)	− 1.26 (1.37)
	Institutional formation (dummy)	0.37** (0.15)	0.09*** (0.03)	0.39 (0.15)
	Constant	− 6.73 (5.05)	8.13*** (0.98)	− 5.60 (5.27)
	Observations	2457	2457	2457 −
	*R* squared	0.02	0.67	0.02
	*F*-test/Wald c2	51.27 (0.00)	15.44 (0.00)	8.17 (0.00)
	Hausman test	2.95(0.81)	67.97 (0.00)	32.69 (0.00)

Dependent variables: 1 knowledge codification, 2 vertical integration, 3 absorptive capacity

Standard errors in parenthesis, ***p < 0.01, **p < 0.05, *p < 0.1

Hypothesis 2 posited that firms would accord higher importance to gaining absorptive capacity in the institutional formation period (2007–2014). The results in Table 6 suggest that institutional formation leads Russian firms to recognise the importance of improving absorptive capacity through an emphasis on human capital. Spending on absorptive capacity in the institutional formation context appears to be higher and more significant—the coefficient being *β* = 5.06 (*p* < 0.10) versus that of *β* = 4.11 in the institutional voids context—which supports hypothesis 2. A swathe of deals in the pharmaceutical sector, whereby Western multinational companies shifted production and some R&D activity to Russia, exploited the country’s vast pool of knowledgeable scientists willing to work at very competitive wage rates (Balashov 2012; Economist 2011). The rapid development of information and communications technology to solve radically new scientific problems such as deciphering DNA likely needed a bevy of information technology professionals proficient in science and engineering (Gokhberg 2004). Large multinational companies, including IBM and Microsoft, made their corporate research laboratories more specialised, entering into various cooperative relationships in the form of contracts with universities and research centres; recruiting specialised researchers; and engaging in consulting, training and other services (Puffer et al. 2016). Once the system was reformed and opened up to global influences, the specific elements within the Russian human capital stock that had spawned a level of ‘ingeniousness’ on the part of specialists could now be turned towards profitable activities (Katkalo and Mowery 1996). The Russian military-industrial complex resulted in the development of a range of technological capabilities and specific and high-tech products (e.g., aircraft, extra-hard metals, special alloys) through highly skilled Russian scientists and engineers (Dyker 2001; Filippov 2011). Such innovation resulted in the development of a specific array of technologies that subsequently proved to be commercially viable in the second period of transition.

Hypothesis 3 posited that firms would accord higher importance to vertical integration in the institutional formation period (2007–2014). The results in Table 7 appear to suggest institutional formation encourages vertical integration, with the coefficient being *β* = 0.11 in the formation phase versus that of *β* = 0.02 (*p* < 0.01) in the institutional voids phase. In the Russian case, modernisation and compliance with GMP (good manufacturing practice) standards as part of the requirements for World Trade Organization membership led many companies to reduce volatilities in quality from suppliers by producing a greater amount of value added in-house (Jensen et al. 2006). The need to control quality and protect proprietary advantages (and the associated rise in the importance of some related technology) may lead a dominant enterprise to devote more significant investment towards in-house integration rather than outsourcing (Gereffi 2001; Dorobantu et al. 2017). Furthermore, direct communication between input and output producers and central coordination of decisions within the firm achieves superior results to those obtainable from the market mechanism, being able to set the price of the input and determine the production amount and attributes, production schedules of each input producer and purchase amount of each output producer (Baumol 1997; Doh et al. 2017).

The robustness test in Table 7 provides the results of panel regressions, incorporating a fixed effects model. The inter-temporal effect of the specific institutional formation (dummy) variable has a significant and positive influence on knowledge codification (*β* = 0.37, *p* < 0.05) and vertical integration (*β* = 0.09, *p* < 0.01). These results corroborate the plausibility of hypotheses 1 and 3 about the positive impact on firm resource allocation and strategic choices. However, more interesting is that scientific output has a significant and positive influence on knowledge codification (*β* = 2.54, *p* < 0.10), vertical integration (*β* = 1.07, *p* < 0.01) and absorptive capacity *β* = 2.40, *p* < 0.10). The effect of domestic spending on R&D also has a positive influence on vertical integration (*β* = 0.67, *p* < 0.01) but not on knowledge codification or absorptive capacity. FDI does not show any effect on any of the dependent variables—which is not altogether unexpected, given that FDI, the standard proxy for institutional quality, may serve to increase multinational, not local Russian investments, the latter being the subject of our analysis.

Overall, the results suggest that institutional formation during the limited window of assessment in this study (2007–2014) appears to have encouraged firms to investment in physical and human capital and technological accumulation and provided stimuli for technical change. Institutional formation reversed the ailing science and technology sectors and instilled confidence in domestic manufacturers that sustainable competitive advantage requires innovative products.

There is general agreement that the unsteady record of reforms that followed the shock therapy transition in the 1990s culminated in numerous institutional voids in Russia (Helmke and Levitsky 2004) and did not inspire business confidence. Drastic institutional shifts cause uncertainty, which leads to opportunistic short-term pursuits such as arbitrage and trading to satisfy immediate demand rather than solid firm-building activities such as investment in vertical integration, knowledge accumulation and human capital. Institutional volatility compels agents to keep investments minimal, leading to a lack of innovation by firms. In the absence of enforced formal institutions and lack of property right protection, firms and other bodies created informal ties that provided some sense of security and legitimisation of transactions (Estrin and Prevezer 2011; Klarin and Sharmelly 2021). In the Russian case, de facto decentralisation led to considerable local resistance in some regions to market reform, splashing the political map with large areas of unreformed institutions (Granville and Leonard 2010). The rapid changes created a hostile environment, where firms were forced to navigate in-between corrupt politicians, organised criminal syndicates and rapidly changing markets. This led to short-termism and distrust; firms abstained from long-term growth investments, such as R&D and modernisation (Radosevic 2003; Filippov 2011).

Our study suggests the importance of paying heed to the complex network of institutions that guide market forces and recognise the necessity for a strong regulatory and enforcement environment. Perhaps, policymakers and business practitioners need to appreciate that successful catch-up and development depend on a robust national innovation system created from strong cooperation by business organisations and the nation-state. Leading organisations are likely to invest heavily in R&D. The ‘Concept of Long-term Socio-Economic Development of the Russian Federation up to the year 2020’, Skolkovo cluster development, university involvement in innovation and knowledge creation, and successive programmes are designed to lay the groundwork for further innovative development, competitiveness based on technology, structural diversification of the economy and modernisation of the infrastructure sectors (Gerasimenko 2012; Skhvediani and Sosnovskikh 2020). It is, however, unclear whether Russia can create an innovation-based economy in the foreseeable future, given the uncertain political climate in recent years. We reiterate that we have not tracked current levels of innovation in Russian firms, and hence, it is impossible to say with any confidence if progress has continued beyond 2014.

## Conclusion and Implications

The chaotic institutions of the 1990s brought about by the fall of the Soviet Union contributed significantly to the decline of industrial production and innovation in Russia (Williams 2011). The ensuing chaos influenced policymakers in Russia to move towards guided market capitalism (Schuman 2011) through renationalising key sectors and import substitution programmes. We have to note that a centralised state has inherent issues, including power structures, lower transparency, political capture by oligarchs and lack of state accountability—all of which inevitably put pressures on businesses. For an exhaustive account of the ill effects of excessive state overreach, see, for example Ledeneva (2013).

Yet, the idea that markets self-regulate, institutions naturally arise in response to market signals and no externalities or distortions need to be addressed (Lall 1996) is debatable. Most Asian economies do not conform to this dictum; their experience shows that they first established non-market institutions before liberalising their economies. The Asian comparative advantage drew on interventions nurturing and creating innovation and export orientation—reducing transaction costs and information asymmetries along the way (Amsden 1997; Lee and Mathews 2010). Such was also the case in China, which undertook very gradual and sequential liberalisation of industries. In the final analysis, markets operate efficiently only when rules, norms and practices are formalised through balancing various forces and non-market institutions.

This research aimed to demonstrate what strategic choices Russian firms made during two phases of transition (1999–2006 and 2007–2014). Investments into knowledge development and codification, absorptive capacity and vertical integration of operations were used as metrics of strategic choices. Taken together, investments in these variables demonstrate the long-term orientation of firms, which is evidenced in this study. The study shows that a lack of investments in intellectual property, human resources, capital equipment and other tangible and intangible firm resources point to short-termism during the 1990s shock therapy transition, as opposed to increased investments into long-term orientation during the state-guided institutional formation period from 2007–2014. For now, our study is one of the few large-scale studies based on available data of publicly listed Russian firms and their strategic choices. As such, the study is a valuable contribution to knowledge about the Russian context, the literature on institutional transition and its impact on firm behaviour during dichotomous transition phases.

For policymakers in transition economies, this study’s conclusions perhaps draw a cautionary tale on the wisdom of wholesale and sudden liberalisation. If anything, the study’s evidence points to the imperatives of building a solid foundation of formal institutions before privatising and liberalising markets. The Russian shock therapy ‘big bang’ approach based on liberalisation, privatisation and deregulation as the pillars of their radical reform strategy lacked an appreciation of the ‘conditions required for it to work effectively’ (Stiglitz 2000). The chaos of an economic transition did not allow economic actors to make sufficient sense of their situation such that they could act coherently (Puffer et al. 2010). The unpredictable new institutional structure did little to regulate corporate governance, accounting procedures, laws on disclosure and payment of dividends. The sheer short-termism among firms in the institutional voids period (Trifilova et al. 2013; Radosevic 1998) curtailed effective development (Yegorov 2009). Exploitative practices and unfair compensation schemes by ruling elites running businesses (Guriev and Rachinsky 2005) and engaging in a barter economy substituting monetary with non-monetary transactions (Rutland 2013; Kogut and Spicer 2002) compounded these issues.

The core limitation of this study is its singular analysis of one transition economy. Going forward, a more in-depth research data analysis is required to expand our knowledge on the impact of institutional changes on strategic choices made by organisations in specific sectors in the more recent era (post-2015) and other emerging economies. This area is vast and under-researched due to several factors, including the lack of transparency and available data on transition economies. Furthermore, this study does not compare the impact of institutional formation under the regime of sanctions that was introduced post the annexation of Crimea in 2014. The study, thus, recommends further research into the impact of institutional formation under the regime of sanctions on firm development. Nonetheless, it is anticipated that understanding the effects of pervasive institutional shifts in Russia is valuable for those researching institutional changes and their effects on organisations in Russia and other countries. This study may also be instructive for countries that have undergone similar transition paths, including the other former Soviet states and other developing countries.

## Appendix. Note on econometric methodology

DA constructs discriminant functions that involve fitting linear discriminant score functions based on observed data concerning several discriminating variables for individual cases whose group membership is known. A typical DA performs a MANOVA or multivariate test of differences between groups on the explanatory variables. The groups are usually best separated by the set of variables whose centroids have maximum distances between them. Thus, if the available variables are *x*_1_, *x*_2_, *x*_3_,…,*x*_*n*_, the best set of variables is the one that will produce maximum distance in space among the group centroids ¯*X*_1_, ¯*X*_2_, ¯*X*_3_, .... ¯*X*_*n*_. If a significant difference among the groups is found, DA then finds which linear combinations of the explanatory variables (independent predictor variables) give the greatest means differences between the existing groups to best discriminate between the groups defined. The classification functions can then estimate the probability of a particular firm’s membership in each group. The equality of group means test is conducted via Wilks’ Lambda, which indicates how well each function separates cases into groups. Finally, DA calculates the percentage of cases correctly classified—through a classification matrix—which confirms/refutes whether the model used on the basis of a priori groupings is accurate. The matrix is reported in a separate table (see Table 4). Fisher’s discriminant function analysis has been extensively used in the economics and management literature (Arya et al. 2000; Lim et al. 2006) where the research problem essentially entails discriminating between two different entities. This technique usually involves fewer violations of assumptions, is robust, handles categorical and continuous variables, and has coefficients that many find easier to interpret (Hair et al. 1998).

In this study, the SPSS package employed for the DA used a stepwise procedure to select the variables for strategic choices to build a model of discrimination. The stepwise procedure is designed to develop the best one-variable model, followed by the best two-variable model, and so forth, until no other variables meet the desired selection rule.

## References

[CR1] Acemoglu Daron, Robinson James A (2002). Economic Backwardness in Political Perspective. American Political Science Review.

[CR2] Adelman, M. A. 1955. Concept and statistical measurement of vertical integration. In *Business Concentration and Price Policy* (pp. 281–330). Princeton University Press.

[CR3] Amsden Alice H (1997). Editorial: Bringing Production Back in — Understanding Government’s Economic Role in Late Industrialization. World Development.

[CR4] Andreeva Tatiana, Garanina Tatiana (2016). Do All Elements of Intellectual Capital Matter for Organizational Performance? Evidence from Russian Context. Journal of Intellectual Capital.

[CR5] Andreoni Antonio, Chang Ha Joon (2019). The Political Economy of Industrial Policy: Structural Interdependencies, Policy Alignment and Conflict Management. Structural Change and Economic Dynamics.

[CR6] Andreoni Antonio, Chang Ha Joon, Scazzieri Roberto (2019). Industrial Policy in Context: Building Blocks for an Integrated and Comparative Political Economy Agenda. Structural Change and Economic Dynamics.

[CR7] Annushkina Olga E (2013). Foreign Market Selection by Russian MNEs – Beyond a Binary Approach?. Critical Perspectives on International Business.

[CR8] Arya Anil, Fellingham John C, Glover Jonathan C, Schroeder Douglas A, Strang Gilbert (2000). Inferring Transactions from Financial Statements. Contemporary Accounting Research.

[CR9] Aslund Anders (2004). Russia’s Economic Transformation Under Putin. Eurasian Geography and Economics.

[CR10] Aslund Anders (2009). Why Market Reform Succeeded and Democracy Failed in Russia. Social Research.

[CR11] Baccini Leonardo, Li Quan, Mirkina Irina, Johnson Kristina (2018). Regional Competition, Business Politicians, and Subnational Fiscal Policy. Business and Politics.

[CR12] Balashov, A.I. 2012. *Formirovanie Mekhanizma Ustoichivogo Razvitiya Farmatsevticheskoi Otrasli: Teoriya i Metodologiya*. Edited by E.A. Tkachenko and S.K. Shwets. Saint-Petersburg: Saint-Petersburg State University of Economics and Finance.

[CR13] Balassa Bela (1988). The Lessons of East Asian Development: An Overview. Economic Development and Cultural Change.

[CR14] Bandarenka Darya (2016). Vertical Integration and Internationalization Strategies of Russian Oil Companies.

[CR15] Baumol William J (1997). Musings on Vertical Integration. International Journal of Social Economics.

[CR16] Bharadwaj Anandhi S (2000). A Resource-Based Perspective on Information Technology Capability and Firm Performance: An Empirical Investigation. MIS Quarterly.

[CR17] Black Bernard S, Tarassova Anna S (2003). Institutional Reform in Transition: A Case Study of Russia. Supreme Court Economic Review.

[CR18] Boubakri, Narjess, Jean-Claude Cosset, and Walid Saffar. 2013. The Role of State and Foreign Owners in Corporate Risk-Taking: Evidence from Privatization. *Journal of Financial Economics* 108(3): 641–58. http://www.sciencedirect.com/science/article/pii/S0304405X1200253X.

[CR19] Braguinsky Serguey, Myerson Roger (2007). A Macroeconomic Model. Economics of Transition.

[CR20] Bresnahan Timothy F, Brynjolfsson Erik, Hitt Lorin M (2002). Information Technology, Workplace Organization, and the Demand for Skilled Labor: Firm-Level Evidence. The Quarterly Journal of Economics.

[CR21] Brocas Isabelle (2003). Vertical Integration and Incentives to Innovate. International Journal of Industrial Organization.

[CR22] Chadee Doren, Roxas Banjo (2013). Institutional Environment, Innovation Capacity and Firm Performance in Russia. Critical Perspectives on International Business.

[CR23] Chang Ha-Joon (2002). Breaking the Mould: An Institutionalist Political Economy Alternative to the Neoliberal Theory of the Market and the State. The Journal of Developing Areas.

[CR24] Chang, Ha-Joon. 2002b. *Kicking Away the Ladder: Development Strategy in Historical Perspective*. Anthem Press.

[CR25] Cheloukhine Serguei, King Joseph (2007). Corruption Networks as a Sphere of Investment Activities in Modern Russia. Communist and Post-Communist Studies.

[CR26] Chen Hai Ming, Lin Ku Jun (2004). The Role of Human Capital Cost in Accounting. Journal of Intellectual Capital.

[CR27] Cirillo Valeria, Sostero Matteo, Tamagni Federico (2017). Innovation and Within-Firm Wage Inequalities: Empirical Evidence from Major European Countries. Industry and Innovation.

[CR28] Cohen WM, Levinthal DA (1990). Absorptive Capacity: A New Perspective on Learning and Innovation. Administrative Science Quarterly.

[CR29] Dakhli Mourad, De Clercq Dirk (2004). Human Capital, Social Capital, and Innovation: A Multi-Country Study. Entrepreneurship & Regional Development.

[CR30] De Supriyo, Dutta Dilip (2007). Impact of Intangible Capital on Productivity and Growth: Lessons from the Indian Information Technology Software Industry. Economic Record.

[CR31] Delios Andrew, Beamish Paul W (2001). Survival and Profitability: The Roles of Experience and Intangible Assets in Foreign Subsidiary Performance. Academy of Management Journal.

[CR32] Doh Jonathan, Rodrigues Suzana, Saka-Helmhout Ayse (2017). International Business Responses to Institutional Voids. Journal of International Business Studies.

[CR33] Dorobantu Sinziana, Kaul Aseem, Zelner Bennet (2017). Nonmarket Strategy Research Through the Lens of New Institutional Economics: An Integrative Review and Future Directions. Strategic Management Journal.

[CR34] Dunning, John H, and Sarianna M Lundan. 2008. “Multinational Enterprises and the Global Economy.” Cheltenham: Edward Elgar Publishing. http://unsw.eblib.com/patron/FullRecord.aspx?p=338808.

[CR35] Dyker David A (2001). Technology Exchange and the Foreign Business Sector in Russia. Research Policy.

[CR36] Economist The (2011). Adventures in Capitalism. The Economist.

[CR37] Eisenhardt, Kathleen M., and Jeffrey Martin. 2000. Dynamic capabilities: what are they? *Strategic Management Journal 21 (10-11): *1105–1121.

[CR38] Estrin Saul, Prevezer Martha (2011). The Role of Informal Institutions in Corporate Governance: Brazil, Russia, India, and China Compared. Asia Pacific Journal of Management.

[CR39] Ettlie, John E., and Ernesto M. Reza. 1992. Organizational Integration and Process Innovation. *Academy of Management Journal* 35 (4): 795–827.

[CR40] Ettlinger Nancy, Hartmann Christopher D (2015). Post/Neo/Liberalism in Relational Perspective. Political Geography.

[CR41] Evensky Jerry (2005). Adam Smith’s Moral Philosophy: A Historical and Contemporary Perspective on Markets, Law, Ethics, and Culture.

[CR42] Feinson, Stephen. 2003. National innovation systems overview and country cases. In: Knowledge Flows and Knowledge Collectives: Understanding The Role of Science and Technology Policies in Development. *Rockefeller Foundation*, Section 1: 13–38. https://cspo.org/legacy/library/110215F7ST_lib_KnowledgeFlowsVo.pdf#page=13. Accessed 4 Nov 2022.

[CR43] Fidrmuc Jarko, Gundacker Lidwina (2017). Income Inequality and Oligarchs in Russian Regions: A Note. European Journal of Political Economy.

[CR44] Filippov, Sergey. 2011. Innovation and R&D in Emerging Russian Multinationals. *Economics, Management, and Financial Markets* 6 (1): 182–206. http://www.ceeol.com/aspx/getdocument.aspx?logid=5&id=790bc59e38664d0e9628a99a6e18d112.

[CR45] Fioretos, Orfeo, Tulia G. Falleti, and Adam Sheingate. 2016. “Historical Institutionalism in Political Science.” In *The Oxford Handbook of Historical Institutionalism*, edited by Orfeo Fioretos, Tulia G. Falleti, and Adam Sheingate, 3–30. Oxford: Oxford University Press. 10.1093/oxfordhb/9780199662814.013.1.

[CR46] Fortesque Stephen (2006). Russia’s Oil Barons and Metal Magnates: Oligarchs and the State in Transition.

[CR47] Gans-Morse Jordan (2013). Threats to Property Rights in Russia: From Private Coercion to State Aggression. Post-Soviet Affairs.

[CR48] Gerasimenko Darya (2012). Russia’s Commercial Policy, 2008–11: Modernization, Crisis, and the WTO Accession. Oxford Review of Economic Policy.

[CR49] Gereffi Gary (2001). Shifting Governance Structures in Global Commodity Chains, with Special Reference to the Internet. American Behavioral Scientist.

[CR50] Geske Dijkstra A (2000). Trade Liberalization and Industrial Development in Latin America. World Development.

[CR51] Gevorkyan Aleksandr V (2013). Russia’s Economic Diversification Potential: The Untold Story?. International Business: Research, Teaching and Practice.

[CR52] Gevorkyan Aleksandr V (2018). Transition Economies: Transformation, Development, and Society in Eastern Europe and the Former Soviet Union.

[CR53] Gokhberg, Leonid, and Tatyana Kuznetsova. 2011. Strategy 2020: New Outlines of Russian Innovation Policy. *Foresight-Russia* 5 (4): 8–30.

[CR54] Gokhberg, Leonid. 2004. Russia’s National Innovation System and the ‘New Economy. *Problems of Economic Transition* 46 (9): 8–34.

[CR55] Goldman Marshall I (2004). Putin and the Oligarchs. Foreign Affairs.

[CR56] Gort, M. 1962. *Diversification and Integration in American Industry*. NBER Books.

[CR57] Government of the Russian Federation. 2011. “Proekt Strategii Innovacionnogo Razvitiya Rossiiskoi Federacii Na Period Do 2020 Goda.” Moscow, Russia. http://www.economy.gov.ru/minec/activity/sections/innovations/development/doc20111020_1. Accessed 4 Nov 2021.

[CR58] Granville Brigitte, Leonard Carol S (2010). Do Informal Institutions Matter for Technological Change in Russia? The Impact of Communist Norms and Conventions, 1998–2004. World Development.

[CR59] Guriev Sergei, Rachinsky Andrei (2005). The Role of Oligarchs in Russian Capitalism. Journal of Economic Perspectives.

[CR60] Hair Joseph F, Black William C, Babin Barry J, Anderson Rolph E (1998). Multivariate Data Analysis- A Global Perspective.

[CR61] Harrigan Kathryn Rudie (1984). Formulating Vertical Integration Strategies. Academy of Management Review.

[CR62] Helfat Constance E, Campo-Rembado Miguel A (2016). Innovation over Successive Technology Lifecycles. Organization Science.

[CR63] Helmke Gretchen, Levitsky Steven (2004). Informal Institutions and Comparative Politics: A Research Agenda. Perspectives on Politics.

[CR64] Hitt Michael A, David Ahlstrom M, Dacin Tina, Levitas Edward, Svobodina Lilia (2004). The Institutional Effects on Strategic Alliance Partner Selection in Transition Economies: China vs. Russia. Organization Science.

[CR65] Hoskisson Robert E, Eden Lorraine, Lau Chung Ming, Wright Mike (2000). Strategy in Emerging Economies. Academy of Management Journal.

[CR66] Inkinen Henri (2015). Review of Empirical Research on Intellectual Capital and Firm Performance. Journal of Intellectual Capital.

[CR67] Jensen Jesper, Rutherford Thomas, Tarr David (2006). The Importance of Telecommunications Reform in Russia’s Accession to the WTO. Eastern European Economics.

[CR68] Katkalo, Valery, Mowery, David C. 1996: Institutional Structure and Innovation in the Emerging Russian Software Industry; in: Mowery, David C.; The International Computer Software Industry; Oxford University Press; New York, Oxford; 240–271.

[CR69] King Lawrence (2002). Postcommunist Divergence: A Comparative Analysis of the Transition to Capitalism in Poland and Russia. Studies in Comparative International Development.

[CR70] Klarin Anton, Ray Pradeep Kanta (2019). Political Connections and Strategic Choices of Emerging Market Firms: Case Study of Russia’s Pharmaceutical Industry. International Journal of Emerging Markets.

[CR71] Klarin Anton, Ray Pradeep Kanta (2021). Industrial Modernisation Through Institutional Upheaval in a Transition Economy. International Journal of Emerging Markets.

[CR72] Klarin Anton, Sharmelly Rifat (2021). Strategic Sensemaking and Political Connections in Unstable Institutional Contexts. Journal of Management Inquiry.

[CR73] Klarin, Anton. 2018. Strategic Choices and Innovation in a Turbulent Institutional Environment: Russian Firms in Transition. 10.26190/unsworks/3488, (accessed 04/11/2022).

[CR74] Knack Stephen, Keefer Philip (1995). Institutions and Economic Performance: Cross-Country Tests Using Alternative Institutional Measures. Economics & Politics.

[CR75] Kogut Bruce, Spicer Andrew (2002). Capital Market Development and Mass Privatization Are Logical Contradictions: Lessons from Russia and Czech Republic. Industrial and Corporate Change.

[CR76] Krueger, Anne O. 1990. The Relationship Between Trade, Employment, and Development. In *The State of Development Economics: Progress and Perspectives*. Cambridge: Basil Blackwell.

[CR77] Krugman Paul (1994). The Myth of Asia’s Miracle. Foreign Affairs.

[CR78] Kumar, N. 1991. Mode of rivalry and comparative behaviour of multinational and local enterprises: The case of Indian manufacturing. *Journal of Development Economics* 35(2): 381–392. 10.1016/0304-3878(91)90056-2.

[CR79] Kumpe Ted, Bolwijn Piet T (1988). Manufacturing: The New Case for Integration. Harvard Buisness Review.

[CR80] Kvintradze, Eteri. 2010. Russia’s Output Collapse and Recovery: Evidence from the Post-Soviet Transition. WP/10/89. *IMF Working Paper*. https://www.imf.org/external/pubs/ft/wp/2010/wp1089.pdf, (accessed 04/11/2021).

[CR81] Lall Sanjaya (1994). The East Asian Miracle: Does the Bell Toll for Industrial Strategy?. World Development.

[CR82] Lall Sanjaya (2006). Some Insights to Reinvent Industrial Strategy in Developing Countries. International Journal of Technology Management.

[CR83] Lall Sanjaya (2013). Reinventing Industrial Strategy: The Role of Government Policy in Building Industrial Competitiveness. Annals of Economics and Finance.

[CR84] Lall, Sanjaya. 1996. Paradigms of development: The East Asian Debate, Oxford Development Studies 24 (2): 111–131.

[CR85] Ledeneva Alena (2013). Can Russia Modernise?: Sistema.

[CR86] Lee Keun, Mathews John A (2010). From Washington Consensus to BeST Consensus for World Development. Asian-Pacific Economic Literature.

[CR87] Lee Sunkee, Meyer-Doyle Philipp (2018). How Performance Incentives Shape Individual Exploration and Exploitation: Evidence from Micro-Data. Organization Science.

[CR88] Laffer, A. B. 1969. Vertical integration by corporations, 1929-1965.* The Review of Economics and Statistics* 51 (1): 91–93.

[CR89] Lim Lewis K S, Acito Frank, Rusetski Alexander (2006). Development of Archetypes of International Marketing Strategy. Journal of International Business Studies.

[CR90] Lundvall Bengt-Åke (2016). The Learning Economy and the Economics of Hope.

[CR91] Macher Jeffrey T, Richman Barak D (2008). Transaction Cost Economics: An Assessment of Empirical Research in the Social Sciences. Business and Politics.

[CR92] Maddigan Ruth J (1981). The Measurement of Vertical Integration. The Review of Economics and Statistics.

[CR93] Mahoney James, Thelen Kathleen, Mahoney James, Thelen Kathleen (2010). A Theory of Gradual Institutional Change. Explaining Institutional Change: Ambiguity, Agency, and Power.

[CR94] Mahoney Joseph T, Rajendran J, Pandian. (1992). The Resource-Based View Within the Conversation of Strategic Management. Strategic Management Journal.

[CR95] Marangos, John. 2003. Was Shock Therapy Really a Shock?. *Journal of Economic Issues* 37(4): 943–66.

[CR96] Matthyssens Paul, Vandenbempt Koen, Berghman Liselore (2006). Value Innovation in Business Markets: Breaking the Industry Recipe. Industrial Marketing Management.

[CR97] McCarthy Daniel J, Puffer Sheila M (2016). Institutional Voids in an Emerging Economy. Journal of Leadership & Organizational Studies.

[CR98] McCarthy Daniel J, Puffer Sheila M, Graham Loren R, Satinsky Daniel M (2014). Emerging Innovation in Emerging Economies: Can Institutional Reforms Help Russia Break Through Its Historical Barriers?. Thunderbird International Business Review.

[CR99] McFaul Michael (1995). State Power, Institutional Change, and the Politics of Privatization in Russia. World Politics.

[CR100] McGuirk Helen, Lenihan Helena, Hart Mark (2015). Measuring the Impact of Innovative Human Capital on Small Firms’ Propensity to Innovate. Research Policy.

[CR101] Medvedev, Dmitry. 2017. “Meeting of the Board of Trustees of the Foundation for the Development of New Technologies, Development and Commercialisation Centre.” The Russian Government. 2017. http://government.ru/en/news/28680/, (accessed 04/11/2021).

[CR102] Michailova Snejina, Worm Verner (2003). Personal Networking in Russia and China: Blat and Guanxi. European Management Journal.

[CR103] Ministry of Industry and Trade of the Russian Federation. 2017. “The List of State Programs.” State Programs. 2017. http://minpromtorg.gov.ru/activities/state_programs/list/, (accessed 04/11/2021).

[CR104] Montanari R (1979). Strategic Choice: A Theoretical Analysis. Journal of Management Studies.

[CR105] Murphy Kevin M, Shleifer Andrei, Vishny Robert W (1992). The Transition to a Market Economy: Pitfalls of Partial Reform. The Quarterly Journal of Economics.

[CR106] Nahapiet, J., & Ghoshal, S. 1998. Social capital, intellectual capital, and the organizational advantage. *Academy of Management Review* 23 (2): 242–266.

[CR107] Nelson Richard R (2008). Economic Development from the Perspective of Evolutionary Economic Theory. Oxford Development Studies.

[CR108] Nelson Richard R, Nelson Katherine (2002). Technology, Institutions, and Innovation Systems. Research Policy.

[CR109] Nelson Richard R, Winter Sidney G (1982). An Evolutionary Theory of Economic Change.

[CR110] Newman, Karen L. 2000. Organizational Transformation During Institutional Upheaval. *Academy of Management Review* 25 (3): 602–19.

[CR111] North Douglass Cecil, Calvert Randall, Eggertsson Thrainn (1990). Institutions, Institutional Change and Economic Performance - Political Economy of Institutions and Decisions. Cambridge.

[CR112] Nyberg AJ, Moliterno TP, Hale D, Lepak DP (2014). Resource-Based Perspectives on Unit-Level Human Capital: A Review and Integration. Journal of Management.

[CR113] OECD. 2011. OECD Reviews of Innovation Policy: Russian Federation 2011.10.1787/9789264113138-en.

[CR114] Osipian A (2018). The Political Economy of Corporate Raiding in Russia.

[CR115] Peng Mike W (2001). How Entrepreneurs Create Wealth in Transition Economies. Academy of Management Executive.

[CR116] Peng, Mike W. 2003. Institutional Transitions and Strategic Choices. *Academy of Management Review* 28 (2): 275–96.

[CR117] Pisano Gary P (1991). The Governance of Innovation: Vertical Integration and Collaborative Arrangements in the Biotechnology Industry. Research Policy.

[CR118] Pratikto Rulyusa (2013). Dynamics of Indonesia’s International Trade a VAR Approach. Procedia Economics and Finance.

[CR119] Puffer Sheila M, McCarthy Daniel J (2011). Two Decades of Russian Business and Management Research: An Institutional Theory Perspective. Academy of Management Perspectives.

[CR120] Puffer Sheila M, McCarthy Daniel J, Boisot Max (2010). Entrepreneurship in Russia and China: The Impact of Formal Institutional Voids. Entrepreneurship Theory and Practice.

[CR121] Puffer Sheila M, McCarthy Daniel J, Jaeger Alfred M (2016). Institution Building and Institutional Voids. International Journal of Emerging Markets.

[CR122] Radosevic Slavo (1998). The Transformation of National Systems of Innovation in Eastern Europe: Between Restructuring and Erosion. Industrial and Corporate Change.

[CR123] Radosevic Slavo (2003). Patterns of Preservation, Restructuring and Survival: Science and Technology Policy in Russia in Post-Soviet Era. Research Policy.

[CR124] Ray Sangeeta (2013). Innovation Strategy in Emerging Market Firms in Response to Institutional Transition Under TRIPS: The Case of the Indian Pharmaceuticals Industry.

[CR125] Ray Sangeeta, Ray Pradeep Kanta (2021). Innovation Strategy of Latecomer Firms Under Tight Appropriability Regimes: The Indian Pharmaceuticals Industry. Journal of International Management.

[CR126] Ray Pradeep Kanta, Ida Masahiro, Suh Chung-Sok, Rhaman Shams-ur (2004). Dynamic Capabilities of Japanese and Korean Enterprises and the ‘Flying Geese’ of International Competitiveness. Asia Pacific Business Review.

[CR127] Robertson Paul L, Langlois Richard N (1995). Innovation, Networks, and Vertical Integration. Research Policy.

[CR128] Rumelt, R. P. 1986.* Strategy, structure and economic performance*. Harvard Business School Press.

[CR129] Rutland Peter (2013). Neoliberalism and the Russian Transition. Review of International Political Economy.

[CR130] Sachs Jeffrey (1991). Crossing the Valley of Tears in East European Reform. Challenge.

[CR131] Sachs Jeffrey, Woo Wing Thye, Fischer Stanley, Hughes Gordon (1994). Structural Factors in the Economic Reforms of China, Eastern Europe, and the Former Soviet Union. Economic Policy.

[CR132] Schuman, Michael. 2011. “State Capitalism vs the Free Market: Which Performs Better?” Time. 2011. http://business.time.com/2011/09/30/state-capitalism-vs-the-free-market-which-performs-better/, (accessed 04/11/2021).

[CR133] Sigurdson, Jon. 2000. Knowledge Creation and Innovation in Geographically Dispersed Organization. *Asia Pacific Journal of Management* 17 (2): 297–330.

[CR134] Silvestre Bruno S (2015). Sustainable Supply Chain Management in Emerging Economies: Environmental Turbulence, Institutional Voids and Sustainability Trajectories. International Journal of Production Economics.

[CR135] Skhvediani Angi, Sosnovskikh Sergey (2020). What Agglomeration Externalities Impact the Development of the Hi-Tech Industry Sector? Evidence from the Russian Regions. International Journal of Technology.

[CR136] Sosnovskikh Sergey (2017). Industrial Clusters in Russia: The Development of Special Economic Zones and Industrial Parks. Russian Journal of Economics.

[CR137] Sosnovskikh Sergey, Cronin Bruce (2021). The Effects of Culture, Attitudes and Perceptions on Industrial Cluster Policy: The Case of Russia. Competition and Change.

[CR138] Stigler George J (1988). Chicago Studies in Political Economy.

[CR139] Stiglitz, J. E. 1999. More instruments and broader goals: Moving toward the post-Washington Consensus. *Revista de Economia Política* 19 (1): 94–120.

[CR140] Stiglitz JE (2000). What I Learned at the World Economic Crisis.

[CR141] Subramaniam, M., & Hewett, K. 2004. Balancing standardization and adaptation for product performance in international markets: testing the influence of headquarters subsidiary contact and cooperation. *Management International Review* 44 (2): 171–194.

[CR142] Teece David J (1985). Multinational Enterprise, Internal Governance, and Industrial Organization. The American Economic Review.

[CR143] Teece David J (2014). A Dynamic Capabilities-Based Entrepreneurial Theory of the Multinational Enterprise. Journal of International Business Studies.

[CR144] Thagard P (2000). Coherence in Thought and Action.

[CR145] Tomizawa Aki, Zhao Li, Bassellier Geneviève, Ahlstrom David (2019). Economic Growth, Innovation, Institutions, and the Great Enrichment. Asia Pacific Journal of Management, Forthcoming.

[CR146] Trifilova Anna, Bartlett Dean, Altman Yochanan (2013). Challenges of International Technology Collaboration with Russian R&D Organisations. Critical Perspectives on International Business.

[CR147] Tucker, I. B., & Wilder, R. P. 1977. Trends in vertical integration in the US manufacturing sector. *The Journal of Industrial Economics* 26 (1): 81–94.

[CR148] Tsang, E. W. K. 2002. Acquiring knowledge by foreign partners from international joint ventures in a transition economy: Learning-by-doing and learning myopia.* Strategic Management Journal* 23 (9): 835–854. 10.1002/smj.251

[CR149] Turkulainen Virpi, Kauppi Katri, Nermes Emma (2017). Institutional Explanations: Missing Link in Operations Management? Insights on Supplier Integration. International Journal of Operations & Production Management.

[CR150] Ulrich, Dave. 1998. “Intellectual Capital = Competence x Commitment.” *Sloan Management Review* 39 (2): 15–26.

[CR151] Ulusoy Veysel, Taş Cumhur (2017). On the Effects of Total Productivity Growth of Economic Freedom and Total Resource Rents: The Case of Both Natural Resource Rich and OECD Countries. Theoretical and Applied Economics XXIV.

[CR152] Viktorov Ilja, Abramov Alexander (2022). The Rise of Collateral-Based Finance Under State Capitalism in Russia. Post-Communist Economies.

[CR153] Volkov Vadim (1999). Violent Entrepreneurship in Post-Communist Russia. Europe-Asia Studies.

[CR154] Wang Fangjun, Luying Xu, Zhang Junrui, Shu Wei (2018). Political Connections, Internal Control and Firm Value: Evidence from China’s Anti-Corruption Campaign. Journal of Business Research.

[CR155] Weick, Karl E., and E Whitener. 2005. “Trust and Fairness in International Human Resource Management: An Organizational Support Theory Perspective.” In *Handbook of Research in International Human Resource Management*. Cheltenham: Edward Elgar Publishing.

[CR156] Williams Dina (2011). Russia’s Innovation System: Reflection on the Past, Present and Future. International Journal of Transitions and Innovation Systems.

[CR157] Williamson John (2009). A Short History of the Washington Consensus. Law and Business Review of the Americas.

[CR158] Woldesenbet Kassa (2018). Managing Institutional Complexity in a Transitional Economy: The Legitimacy Work of Senior Managers. International Journal of Emerging Markets.

[CR159] Yegorov Igor (2009). Post-Soviet Science: Difficulties in the Transformation of the R&D Systems in Russia and Ukraine. Research Policy.

[CR160] Yeltsin Boris (2000). Prezidentskii Marafon.

[CR161] Zhang Kevin Honglin, Song Shunfeng (2000). Promoting Exports: The Role of Inward FDI in China. China Economic Review.

